# Pediatric Adenotonsillectomy over 20 Years in a High-Volume Italian Centre: Positive Outcomes with Low Complications—The Sassuolo Hospital Experience

**DOI:** 10.3390/pediatric18020045

**Published:** 2026-03-23

**Authors:** Gennaro Confuorto, Renato Baldi, Elisa Cigarini, Giorgio Di Lorenzo, Silvia Menabue, Federico Spagnolo, Margherita Trani, Massimo Zanni, Livio Presutti, Daniele Marchioni, Paolo Gambelli

**Affiliations:** 1 Department of Otorhinolaryngology, Sassuolo Hospital, 41049 Modena, Italy; r.baldi@ospedalesassuolo.it (R.B.); e.cigarini@ospedalesassuolo.it (E.C.); g.dilorenzo@ospedalesassuolo.it (G.D.L.); s.menabue@ospedalesassuolo.it (S.M.); f.spagnolo@ausl.mo.it (F.S.); m.trani@ospedalesassuolo.it (M.T.); m.zanni@ospedalesassuolo.it (M.Z.); p.gambelli@ospedalesassuolo.it (P.G.); 2 Department of Otorhinolaryngology—Head and Neck Surgery, IRCCS Azienda Ospedaliero-Universitaria di Bologna, Policlinico S. Orsola-Malpighi, 40138 Bologna, Italy; livio.presutti@unibo.it; 3 Department of Otorhinolaryngology—Head and Neck Surgery, University Hospital of Modena, 41125 Modena, Italy; daniele.marchioni@unimore.it

**Keywords:** adenotonsillectomy, postoperative hemorrhage, pediatric otolaryngology, Colorado microdissection needle, observational study

## Abstract

Background: Pediatric adenotonsillectomy is commonly performed for infectious and obstructive indications, but postoperative hemorrhage remains a concern. This study describes outcomes from a high-volume territorial network in southern Modena province, Italy. Methods: Retrospective observational study of 10,753 pediatric patients (aged 3–18 years) undergoing adenotonsillectomy at Sassuolo Hospital and affiliates (Vignola, Pavullo) from 2005 to 2024. Indications included recurrent tonsillitis (Paradise criteria), obstructive sleep apnea (OSA) (polysomnography-confirmed or clinical), and recurrent otitis media or otitis media with effusion (OME). Surgical techniques included curettage adenoidectomy and Colorado microdissection needle tonsillectomy. Our institutional postoperative care protocol included analgesics, oral hydration, soft diet, antibiotics (amoxicillin) and scheduled follow-up; however, no analysis regarding this protocol was intended to demonstrate correlations with study outcomes. Primary outcomes were postoperative hemorrhage (overall and requiring revision), stratified by indication, age, and technique, and contextualized against ranges reported in large published cohorts (qualitative, exploratory comparison). Secondary outcomes included pain (VAS scores), infection rates, and tissue regrowth. Data completeness was verified via electronic records (95.6%). Statistical analyses used descriptive statistics with 95% confidence intervals (95% CI) and inferential tests for within-cohort comparisons (χ^2^ tests, Fisher’s exact test, and *t*-tests where appropriate). Results: A total of 10,753 procedures were analyzed (4325 tonsillectomies, 3942 adenotonsillectomies, 2486 adenoidectomies). Postoperative hemorrhage occurred in 202 patients (1.88%; 95% CI 1.64–2.15%); surgical revision was required in 75 (0.70%; 95% CI 0.56–0.87%), with multifactorial stratification showing higher risk for infectious indications (OR 1.41 vs. OSA), younger age < 5 years (OR 2.1), and tonsillectomy origin (OR 8.25 vs. adenoidectomy); all rates are at the lower end of literature ranges (2–5% and 0.9–2.5%, respectively), in line with large published cohorts, although these comparisons are qualitative and exploratory. Mean VAS pain scores decreased from 3.2 (day 1) to 1.1 (day 7). No significant infections occurred; tissue regrowth rates aligned with the literature (adenoidal 6–26%, tonsillar 5–10%). Conclusions: Sassuolo Hospital’s experience highlights favorable postoperative outcomes and low complication rates in adenotonsillar surgery. Limitations include the retrospective design, potential selection bias and long period evaluation. Prospective studies are needed to confirm these findings.

## 1. Introduction

Adenotonsillectomy is one of the most common surgical procedures in pediatric otolaryngology and is primarily performed to treat chronic adenotonsillitis, obstructive sleep apnea (OSA), recurrent otitis media, and associated hearing loss. These conditions, often caused by hypertrophy of the tonsils and adenoids, can result in substantial morbidity in children, including breathing difficulties, sleep disturbances, recurrent infections, and impaired hearing. Consequently, adenotonsillectomy is frequently recommended to improve both quality of life and overall health outcomes in affected children [1]. Historically, adenotonsillectomy has been performed using a variety of techniques, ranging from traditional cold steel dissection to more modern approaches such as electrocautery and laser surgery. Over time, these techniques have evolved with the aim of shortening operative time, minimizing postoperative pain, and reducing the risk of complications, particularly hemorrhage and infection [1]. In parallel with these surgical advances, improvements in preoperative assessment tools and standardized postoperative care protocols have further contributed to better patient outcomes [2]. In Italy, in the Emilia-Romagna region, the Sassuolo Hospital network (Sassuolo, Vignola, Pavullo affiliates) is notable for its high volume of pediatric adenotonsillectomy procedures. These three institutions form an integrated hospital network that provides public healthcare services across the southern area of Modena province. According to the 2024 National Outcome Program report, this network ranks first in the Emilia-Romagna region and third nationwide for the volume of adenotonsillectomy procedures, despite not being dedicated pediatric centers [3]. This ranking reflects the team’s high procedural volume and commitment to quality. A key element of the success of the pediatric adenotonsillectomy program at Sassuolo, Vignola, and Pavullo hospitals is the use of innovative techniques and advanced technologies, such as the Colorado microdissection needle (Stryker), which has been associated with reduced intraoperative bleeding and improved tissue preservation [4]. In addition, a comprehensive approach to patient management—including thorough preoperative evaluation and robust psychological and multidisciplinary support—has further contributed to favorable clinical outcomes [5]. Despite the high volume of procedures, postoperative complications, particularly hemorrhage, remain a major concern in pediatric adenotonsillectomy. Reports in the literature describe highly variable rates of postoperative bleeding, generally ranging from 2% to 5%, with revision surgery required in approximately 0.9% to 2.5% of cases [6]. The management of these complications requires an experienced surgical team, meticulous technique, and prompt intervention when necessary. The present study aims to evaluate the 20-year experience of the Otorhinolaryngology team at Sassuolo Hospital in performing pediatric adenotonsillectomies, with a focus on surgical outcomes, complication rates, and the integration of innovative surgical techniques. Key clinical uncertainties remain regarding hemorrhagic risk stratification across indications and techniques, age-specific vulnerability patterns, and real-world complication profiles in high-volume territorial networks versus single-center experiences. This study analyzes data from 10,753 procedures across Sassuolo, Vignola, and Pavullo hospitals (2005–2024) to address these gaps, providing insights into current practice effectiveness and real-world outcomes using a standardized multidisciplinary network approach.

## 2. Materials and Methods

### 2.1. Study Design

This retrospective observational study was conducted at Sassuolo Hospital (Modena, Italy) and its affiliated hospitals in Vignola and Pavullo, and we analyzed the outcomes of pediatric adenotonsillectomy procedures performed over a 20-year period (2005–2024). The data completeness was 95.6% (10,753/11,247 patients), achieved through electronic health records (fully implemented since 2010) and digitized paper charts from 2005 to 2009 (verified by 98% completeness audit). Surgical protocols were standardized from study outset in 2005, with routine use of the Colorado microdissection needle (Stryker^®^) as the primary tonsillectomy technique across all participating surgeons and centers. No internal comparator group (e.g., cold steel tonsillectomy or alternative electrosurgical devices) was available, as the Colorado microdissection needle and standardized protocols were adopted uniformly across the study period. The study protocol was approved by the institutional review board of Sassuolo Hospital, and written informed consent was obtained from the legal guardians of all patients prior to surgery.

### 2.2. Patient Selection and Surgical Indications

A total of 10,753 pediatric patients who underwent adenotonsillar surgery at the three participating hospitals during the study period were included in the analysis. Surgical indications were based on established clinical criteria and international guidelines and encompassed both infectious and obstructive conditions. Tonsillectomy (with or without adenoidectomy) was performed in children with (1) recurrent tonsillitis or recurrent throat infections meeting guideline thresholds (Paradise criteria: at least seven well-documented episodes in the preceding year, five episodes per year for two consecutive years, or three episodes per year for three consecutive years, with associated clinical features such as fever, cervical adenopathy, tonsillar exudate, or a positive group A streptococcal test) and/or (2) obstructive sleep-disordered breathing or obstructive sleep apnea (OSA) attributable to tonsillar hypertrophy, particularly when OSA was documented by overnight polysomnography or when significant daytime morbidity (growth failure, behavioral disturbances, or poor school performance) was present.

Adenoidectomy, either alone or in combination with tonsillectomy, was primarily indicated for adenoidal hypertrophy causing nasal obstruction or sleep-disordered breathing, and for otitis media with effusion or recurrent acute otitis media associated with adenoidal disease. In line with current recommendations, adenoidectomy was preferentially considered in children (especially over 4 years of age) with persistent or recurrent otitis media with effusion requiring tympanostomy tubes, in those needing repeat tube insertion, and in patients with chronic nasal obstruction or chronic adenoiditis.

Thus, adenotonsillectomy was most commonly performed in cases of combined adenotonsillar hypertrophy responsible for OSA or mixed obstructive symptoms or in children with chronic or recurrent adenotonsillitis [7]. Children with significant comorbidities, such as severe cardiovascular disease, severe bleeding disorders, or conditions increasing anesthetic risk (ASA ≥ IV), were excluded from surgery and from the present analysis.

Patients were categorized according to the type of procedure performed: adenoidectomy, tonsillectomy, or adenotonsillectomy.

### 2.3. Preoperative Assessment

All patients underwent a standardized preoperative evaluation that included the following:

1. Clinical Evaluation: This included a detailed medical history and physical examination, including assessment of the frequency of infections, severity of obstructive sleep apnea, and hearing impairment.
2. Flexible Fiberoptic Nasopharyngoscopy: This was used to assess the degree of adenoid hypertrophy, graded on a scale of 1 to 4 (grade 1 being minimal and grade 4 representing complete obstruction of the nasopharynx).
3. Audiometric and Tympanometric Assessments: Audiometry was performed to evaluate hearing loss, with behavioral audiometry used for children under 5 years of age. Tympanometry was used to assess middle ear function, particularly in cases of recurrent otitis media [8].
4. Preoperative Blood Work: Routine preoperative tests were conducted to assess the child’s general health and to rule out any contraindications for surgery.

### 2.4. Surgical Procedures

**Figure 1:**
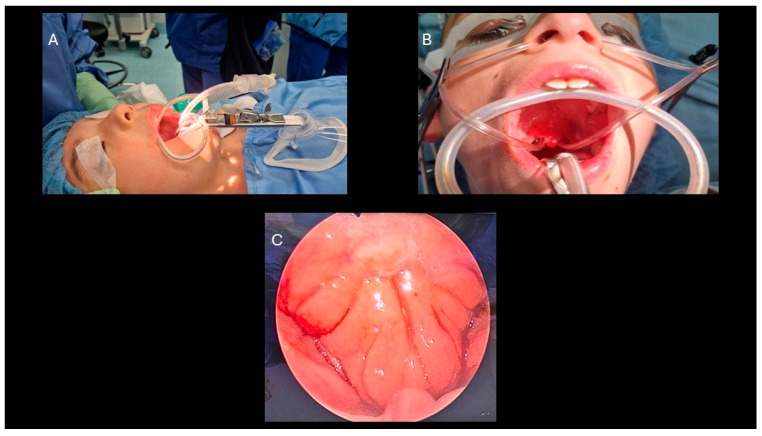
(**A**) Patient positioning for adenotonsillectomy: The head is slightly extended, with a mouth gag in place to maintain oral access and stabilized by an anesthesiologic mask. (**B**) Palatal retraction achieved using two Nelaton nasal catheters retrieved through the oral cavity and secured externally. (**C**) Adenoid visualization with 45° 4 mm endoscope through the oral cavity.

Adenotonsillectomies were performed by a team of experienced otolaryngologists using standardized surgical techniques. Before starting the procedure, the patient is placed under general anesthesia to ensure the child is fully unconscious and pain-free. An oral endotracheal intubation is performed for airway management. Then, the patient is positioned in a supine manner with the head tilted backward to provide optimal access. The Boyle–Davis mouth gag is used to secure the mouth open during the surgery, allowing the surgeon to work with sufficient space. In our team, the mouth gag is held in position during the surgery using a pediatric anesthesia face mask (Figure 1A) [9].

**Figure 2:**
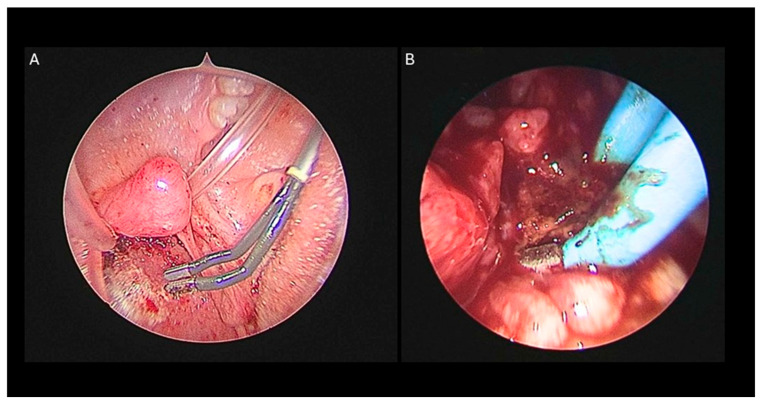
(**A**) Use of a 45° endoscope via the oral route for enhanced hemostatic control of the nasopharyngeal region, facilitated by the retraction of the soft palate using Nelaton catheters. (**B**) Precise cauterization of the bleeding sites in the nasopharynx.

**Figure 3:**
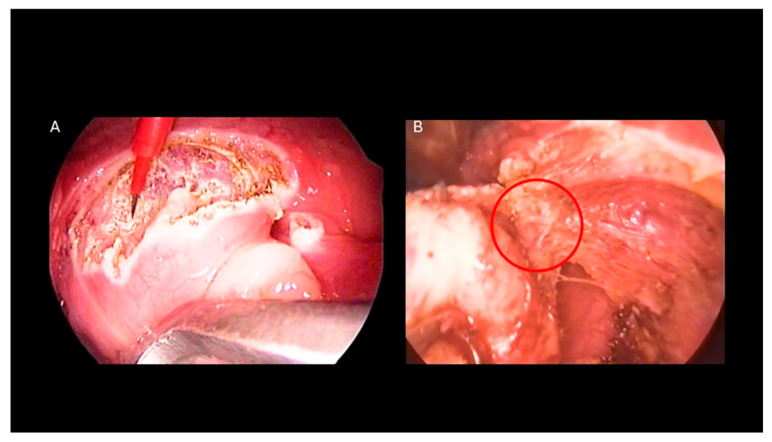
(**A**) Tonsillectomy performed with a Colorado microdissection needle: the dissection is conducted along the plane of the tonsillar capsule, exposing the underlying muscular layer. (**B**) Cauterization of a small remnant of tonsillar tissue at the inferior pole is performed to mitigate the risk of hemorrhage associated with the lingual artery.

1. Adenoidectomy: Adequate retraction of the soft palate was considered essential to ensure full visualization and access to the adenoids. This was achieved using two Nelaton nasal catheters, one placed in each nasal cavity, retrieved through the oropharynx and secured externally with a Klemmer clamp (Figure 1B). Before adenoid removal, a preliminary endoscopic evaluation of the nasopharynx was performed with a 45° 4 mm endoscope introduced through the oral cavity (Figure 1C). Adenoidectomy was carried out using a Negus curette, which allowed gentle curettage of the adenoid tissue from the nasopharyngeal wall. During curettage, minor bleeding from the surgical site was controlled by gentle packing of the nasopharynx with gauze to achieve hemostasis. If bleeding persisted despite manual compression, an endoscopic approach was adopted to allow more precise visualization and control. A 45° 4 mm nasal endoscope was introduced through the mouth to inspect the nasopharynx and identify bleeding vessels, which were then coagulated using diathermy (Figure 2A,B) [10].
2. Tonsillectomy: Tonsillectomy was performed using several techniques, including cold steel dissection, monopolar or bipolar electrocautery, laser dissection, and radiofrequency-based methods. The choice of technique depended on the surgeon’s experience, patient characteristics, and the available equipment. In our center, monopolar electrocautery dissection using the Colorado microdissection needle was the preferred technique. This method uses a high-frequency electrical current to cut and coagulate tissue through a fine tungsten-coated tip (5 µm), allowing precise dissection and controlled thermal spread [9]. The tonsil was dissected by carefully separating it from the surrounding pharyngeal tissues, including the muscular layer, blood vessels, and mucosa, with particular attention given to preserving the palatopharyngeal and levator veli palatini muscles. Hemostasis was achieved with gauze compression and electrocautery of small bleeding vessels. In most cases, sutures were not required in the tonsillar bed, which was left to heal by secondary intention. The technique was designed to minimize thermal injury, preserve the tonsillar pillars, and deliberately leave a small cuff of tissue at the inferior pole, which was then coagulated using bipolar forceps to reduce the risk of bleeding near the tongue base [11].
3. Extracapsular vs. Intracapsular Techniques: Tonsillectomy was performed using either an extracapsular or an intracapsular approach, chosen according to the clinical indication and patient profile. In the extracapsular technique, the tonsil was completely removed, along with its fibrous capsule (Figure 3A,B). In the intracapsular technique, a small portion of tonsillar tissue was intentionally left in situ to facilitate healing and reduce the risk of postoperative hemorrhage. Intracapsular tonsillectomy is associated with lower postoperative bleeding rates, faster recovery, and reduced pain, although it carries a risk of tonsillar regrowth. In accordance with international guidelines, extracapsular dissection was typically preferred for patients with chronic or recurrent tonsillitis refractory to medical therapy, whereas the intracapsular approach was mainly used in children with OSA, for whom complete tonsillar removal is not mandatory, and in patients at increased risk of bleeding or in need of more rapid recovery (e.g., children with Down syndrome) [11].
4. Endoscopic tympanic paracentesis and transtympanic drainage placement are performed in conjunction with adenotonsillar surgery when indicated by audiometric findings. The use of the endoscopic approach allows for enhanced visualization of the tympanic membrane, facilitating the detection of any abnormalities and optimizing the procedure. This technique is aimed at improving middle ear ventilation, thereby addressing underlying dysfunctions and contributing to better postoperative outcomes [8].
5. Postoperative Care: All patients were monitored just after surgery in the post-anesthesia care unit (PACU), and after a period of observation, they went in the Pediatric unit and were discharged according to the type of surgery:
  - Adenoidectomy: Patients were usually discharged on the same day. Postoperative home care included topical antibiotic nasal drops for 7 days and 7 days of home rest.
  - Tonsillectomy/Adenotonsillectomy: Patients were admitted for at least one night of observation. Postoperative management included home rest and adherence to a soft, cool diet for 15 days to promote healing and reduce discomfort [12].

Postoperative care included standardized non-opioid analgesia (acetaminophen and ibuprofen with opioids reserved for severe pain or inadequate response to first-line therapy) oral hydration, a soft diet and scheduled clinical follow-up [13]. During most of the study period, oral amoxicillin (50 mg/kg/day for 5–7 days) was routinely prescribed as postoperative prophylaxis according to local institutional practice, although this approach is not universally recommended in current international guidelines. Postoperative antibiotic use was described as part of routine care but not analyzed for potential roles in study outcomes; therefore, no additional analyses are required.

### 2.5. Complication Monitoring

Postoperative complications, including hemorrhage, infection, and pain, were systematically recorded during the hospital stay and at follow-up visits scheduled 1–2 weeks after surgery. Hemorrhage was classified as minor when controlled with local measures (e.g., observation, topical hemostasis) and major when requiring surgical revision under general anesthesia [14]. The incidence of postoperative hemorrhage was calculated and compared with published data from the literature.

### 2.6. Emergency Support

Sassuolo Hospital maintains a formal collaboration with the University Hospital of Modena, which provides 24 h emergency coverage for postoperative complications. Within the territorial healthcare model, the ENT surgical team at Sassuolo Hospital is on call only for patients hospitalized for surgery on that specific day. In cases of postoperative hemorrhagic complications occurring after discharge, particularly during nighttime hours, patients and their families are instructed to present directly to the Otorhinolaryngology Unit of the University Hospital of Modena, where a 24 h on-call surgical service is available for emergency management.

### 2.7. Statistical Analysis

Descriptive statistics summarized patient demographics, surgical characteristics, and clinical outcomes. Continuous variables (age, operative duration) were reported as means ± standard deviation (SD) or medians (IQR); categorical variables as counts (*n*), percentages (%), and 95% confidence intervals (95% CI) using the Wilson score method. Primary outcomes (postoperative hemorrhage, surgical revision) were stratified by surgical indication (OSA, recurrent tonsillitis, otitis media), age group (3–5, 6–10, >10 years), anatomical origin (adenoidectomy vs. tonsillectomy), and tonsillar technique (extracapsular vs. intracapsular). Comparisons with literature benchmarks were limited to qualitative, descriptive contextualization of our observed rates within published ranges, without formal hypothesis testing against literature-derived values. Inferential analyses within our cohort (e.g., comparisons across indications, age groups, surgical techniques) used chi-squared (χ^2^) tests or Fisher’s exact test (expected cell counts < 5), with effect sizes reported as odds ratios (OR) and 95% CI. Secondary outcomes (pain VAS scores) were analyzed with paired *t*-tests where applicable. Statistical significance was defined as *p* < 0.05 (two-sided). No adjustments for multiplicity were applied given the exploratory nature of subgroup analyses, so the *p*-values for subgroup analyses should be interpreted with caution. All analyses were performed using IBM SPSS Statistics, version 25.0 (IBM Corp., Armonk, NY, USA) [15].

## 3. Results

### 3.1. Surgical Volume and Demographics

Over the 20-year study period (2005–2024), a total of 10,753 pediatric adenotonsillar procedures were performed across Sassuolo, Vignola, and Pavullo hospitals. These included 4325 tonsillectomies (40.2%), 3942 adenotonsillectomies (36.7%), and 2486 adenoidectomies (23.1%). The patient cohort comprised children aged 3–18 years, with a mean age of 6.8 ± 3.1 years. The sex distribution was 56.1% male (*n* = 6032) and 43.9% female (*n* = 4721).

### 3.2. Postoperative Hemorrhage Rates

Of the 10,753 children who underwent adenotonsillar surgery, 202 (1.88%; 95% CI 1.64–2.15%) experienced postoperative bleeding. Most hemorrhages (127/202, 62.9%; 95% CI 56.03–69.24%) were classified as minor and managed conservatively with clinical observation or local tamponade. The remaining 75/202 (37.1%; 95% CI 30.76–43.97%) required more aggressive intervention, including revision surgery under general anesthesia. The overall surgical revision rate was 0.70% (95% CI 0.56–0.87%).

Postoperative hemorrhage was stratified by multiple factors. Among the 75 cases requiring surgical revision, the origin was adenoidectomy-related in 12/75 (16.0%; 95% CI 9.0–26.5%) versus tonsillectomy-related in 63/75 (84.0%; 95% CI 73.5–91.0%). Of tonsillar revisions, 52/63 (82.5%; 95% CI 71.0–90.5%) followed extracapsular tonsillectomy versus 11/63 (17.5%; 95% CI 9.5–29.0%) after the intracapsular technique (*p* = 0.41, Fisher’s exact test) [16] (Table 1).

Hemorrhage Rates by Surgical Indication: The primary indications were obstructive sleep apnea (OSA) in 5215 patients (48.5%), recurrent tonsillitis in 4158 (38.7%), and otitis media with effusion/recurrent otitis media in 1380 (12.8%). Of the 202 hemorrhage cases, 85 occurred in OSA patients (1.63%; 95% CI 1.32–2.02%), 95 in recurrent tonsillitis (2.28%; 95% CI 1.87–2.79%), and 22 in otitis media cases (1.59%; 95% CI 1.04–2.40%). Recurrent tonsillitis showed significantly higher hemorrhage risk versus OSA (OR 1.41; 95% CI 1.05–1.89; *p* = 0.021, χ^2^ test) and those result are aligned with the literature, consistent with chronic inflammation and peritonsillar fibrosis, while otitis media rates were comparable (*p* = 0.78) [17,18]. Age stratification revealed higher revision risk in younger children: age 3–5 years accounted for 38/75 revisions (50.7%; 95% CI 39.1–62.2%) versus 22/75 (29.3%; 95% CI 19.7–40.9%) in 6–10 years and 15/75 (20.0%; 95% CI 12.1–30.8%) in >10 years (OR 2.1 for age 3–5 years vs. >10 years, 95% CI 1.1–4.0; *p* = 0.02). Thus, most revisions were tonsillar in origin (predominantly extracapsular), with independent risks from younger age and infectious indications [19] (Table 2).

### 3.3. Complication Comparison with the Literature

When compared qualitatively with the national and international literature, complication rates at Sassuolo Hospital appear at the lower end of those reported in large cohort studies. Published data describe postoperative hemorrhage rates ranging from 2% to 5%, with surgical revision required in 0.9–2.5% of cases. In our cohort, postoperative hemorrhage was 1.88% (95% CI 1.64–2.15%) and surgical revision was 0.70% (95% CI 0.56–0.87%), towards the lower bounds of the published ranges. These contextual comparisons are descriptive and exploratory only, as literature-derived ranges do not constitute a formal control group and differ in case mix, indications, and follow-up. Potential contributors to our low rates include high procedural volume, standardized techniques, and rigorous postoperative surveillance, although residual confounding cannot be excluded in this observational design [20,21].

No significant postoperative infectious complications were observed despite extensive rhinopharyngeal cauterization in select cases. Early extrusion of transtympanic ventilation tubes occurred in only three cases (exact incidence unavailable); all were successfully repositioned without sequelae.

Regarding tissue regrowth, rates of adenoidal regrowth after adenoidectomy (6–26% in literature) and tonsillar regrowth after tonsillotomy (5–10%, particularly in younger children or those with atopy) in our cohort were consistent with published ranges, with no need for systematic revision surgery [22,23]. The majority of patients experienced mild to moderate postoperative pain. Mean Visual Analog Scale (VAS) scores were 3.2 on postoperative day 1, decreasing to 1.1 by day 7 (*p* < 0.001, paired *t*-test). Only 2.1% of patients (*n* = 226/10,753; 95% CI 1.84–2.41%) required opioid analgesia beyond the first 24 h. Length of hospital stay was 1 day for adenoidectomy and 1–2 days for tonsillectomy/adenotonsillectomy, consistent with high-volume center standards [24].

## 4. Discussion

Adenotonsillectomy remains one of the most frequently performed pediatric surgical procedures worldwide and is typically indicated for chronic tonsillitis, obstructive sleep apnea, recurrent otitis media or effusive otitis media with and associated hearing loss [1]. In this study, the outcomes of 10,753 pediatric adenotonsillar procedures performed over 20 years at three hospitals in the Modena province were analyzed. The data highlight the ability of this territorial network to maintain low complication rates—particularly with regard to postoperative hemorrhage—while implementing innovative surgical techniques designed to improve precision and minimize tissue trauma.

### 4.1. Postoperative Hemorrhage and Surgical Revision

**Figure 4:**
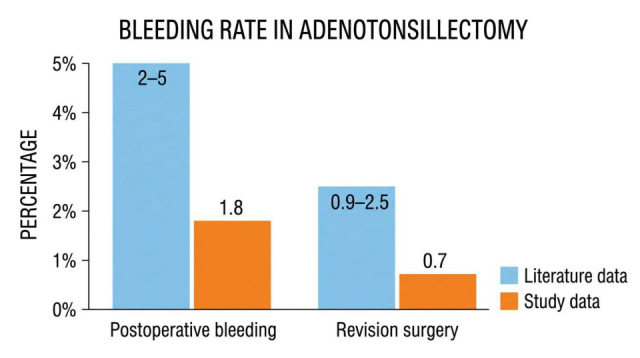
Post-adenotonsillectomy hemorrhage rates and surgical revision rates: comparison between Sassuolo Hospital and the literature.

Postoperative hemorrhage rates after adenotonsillectomy vary widely across studies (2–5% overall, 0.9–2.5% requiring revision), with reported means around 3.5% and 1.7%, respectively. In our cohort, hemorrhage and revision rates were 1.88% (95% CI 1.64–2.15%) and 0.70% (95% CI 0.56–0.87%), respectively, which fall at the lower end of these published ranges. These comparisons with the literature are qualitative and exploratory, given the heterogeneity in patient selection, indications, definitions of hemorrhage, and follow-up across studies, and are not based on formal hypothesis testing against literature means (Figure 4) [16]. Multivariate stratification identified infectious indications (OR 1.41 vs. OSA, *p* = 0.021), younger age < 5 years (OR 2.1, *p* = 0.02), and tonsillectomy origin (OR 8.25 vs. adenoidectomy, *p* < 0.001; 84% of revisions) as key risk factors, reflecting chronic peritonsillar vascularity, tissue immaturity, and tonsillar vascular complexity, respectively. Despite 38.7% recurrent tonsillitis cases—known for hypervascularity—our rates remained at the lower end of published benchmarks, potentially reflecting contributions from standardized protocols and high procedural volume [17]. Stratification by tonsillar technique revealed numerically lower revision rates after intracapsular tonsillectomy compared to extracapsular (17.5% of tonsillar revisions vs. 82.5%; *p* = 0.41), though this difference did not reach statistical significance. This descriptive pattern is consistent with the literature demonstrating reduced postoperative hemorrhage with partial tonsillectomy due to the preservation of the tonsillar capsule and decreased tissue trauma. Our findings are hypothesis-generating; adequately powered comparative studies would be needed to confirm technique-specific effects. Additionally, no adjustments for multiplicity were applied, given the exploratory nature of subgroup analyses (age group, indication, technique, anatomical origin). Consequently, *p*-values from these multiple subgroup comparisons should be interpreted cautiously. The Colorado microdissection needle, used routinely since 2005, may have contributed to addressing these specific risks and is consistent with the low hemorrhage rates observed in our cohort: its ≤0.7 mm thermal spread (vs. 2 mm conventional) minimizes collateral damage in hypervascular tonsils; 5–20 mL blood loss (vs. 30–70 mL) facilitates hemostasis in younger patients; and precision at the inferior pole reduces tonsillar bed bleeding (Table 3). Published data report lower hemorrhage rates with this technique (0.8–1.5% vs. 2–4%) [25,26,27,28]. In our experience, these characteristics could be associated with favorable outcomes, particularly in extracapsular dissections for infectious indications, although causality cannot be inferred from our non-comparative design. Several factors likely contributed, including meticulous hemostasis and structured monitoring; however, as an observational study, causality cannot be established, and prospective comparative trials are needed [16].

### 4.2. Pain Management and Recovery

Postoperative pain is a major determinant of recovery after pediatric adenotonsillectomy. In our cohort, VAS scores systematically recorded by nursing staff (paper charts pre-2015, electronic post-2015; 82.4% completeness, *n* = 8860/10,753) showed mild–moderate pain with rapid decline: mean 3.2 ± 1.9 (day 1) to 1.1 ± 1.1 (day 7) among patients with paired data (*n* = 2150). Only 2.1% (*n* = 226/10,753; 95% CI 1.84–2.41%) required opioids beyond 24 h, numerically lower than literature ranges (3–5%) [13]. This pain trajectory descriptively aligns with our multimodal non-opioid protocol (paracetamol + ibuprofen) and may reflect reduced thermal injury from Colorado microdissection (20–30% lower VAS reported in the literature vs. conventional, Table 3) [25,26,27,28]. Infection rates were negligible, consistent with historical series reporting 1–3% postoperative infections. Tissue regrowth, while not systematically quantified, remained within expected literature ranges: adenoidal regrowth (6–26%) and tonsillar regrowth post-intracapsular tonsillotomy (5–10%, higher in atopics and <4 years) [22,23]. No revision surgeries for regrowth were required, consistent with the selective use of intracapsular technique for OSA cases where complete tonsillar removal is not mandatory. Collectively, these secondary outcomes reinforce the safety profile of the Sassuolo protocol across multiple domains, complementing the primary hemorrhage findings. Retrospectively extracted scores from mixed records introduce potential reporting bias; findings are descriptive rather than definitive. The multimodal approach—integrating technique, pharmacology, and monitoring—likely contributes to comprehensive postoperative recovery, though prospective validation remains essential.

### 4.3. Multidisciplinary and Psychological Support 

An important feature of our model is multidisciplinary team integration (otolaryngologists, anesthesiologists, pediatricians, specialized nursing) with descriptive psychological support initiatives offered perioperatively. Programs such as the “Giocamico” play therapy initiative and “Discovering Planet O.R.” project familiarized children with the operating room environment, reaching 92% of patients (*n* = 9893/10,753). Parental feedback from routine postoperative satisfaction surveys (non-validated, unstructured format; response rate 78.6%, *n* = 8450) indicated the following:

- A total of 94% reported “significantly reduced parental anxiety”;
- A total of 89% noted “improved child cooperation” in the operating room.

Although formal anxiety scales were not measured (these represent descriptive, exploratory clinical impressions rather than validated psychological outcomes), such initiatives may enhance perioperative experience and family satisfaction, though their isolated impact remains hypothesis-generating only.

### 4.4. The Importance of Inter-Hospital Collaboration in Otolaryngology Within Modena Province

The long-standing hub-and-spoke organizational model applied in the Modena province underscores the importance of inter-hospital collaboration in the management of otolaryngological diseases. From the initial outpatient evaluation, cases are triaged according to complexity, ensuring that patients receive care in the most appropriate setting. Complex cases, including oncological and otoneurosurgical conditions, are referred to the tertiary center (the University Hospital of Modena, Policlinico), which functions as the primary referral hub for advanced pathology. Conversely, Sassuolo Hospital coordinates outpatient and surgical activities for the southern area of the province, including Vignola, Pavullo, and Montefiorino, and focuses on medium- to low-complexity conditions. Within this framework, adenotonsillectomy constitutes a major component of surgical activity. Successful management of these patients requires a well-defined preoperative assessment pathway, fully equipped operating rooms, and a dedicated multidisciplinary team. Particular attention is given to postoperative monitoring, especially considering that many children come from geographically diverse areas, including the Apennine region, where access to emergency services may be less immediate. To ensure safe postoperative follow-up, parents receive detailed written and verbal instructions on home surveillance and on how to seek immediate assistance in the event of unexpected symptoms. Discharge documentation systematically includes emergency contact details for on-call physicians. Given the potential severity of postoperative hemorrhage, this risk is thoroughly discussed during the informed consent process, and clear information is provided regarding the pathways for urgent care. Families are explicitly informed that, outside the coverage hours of the Sassuolo otolaryngology unit, they should directly access the University Hospital of Modena, where round-the-clock specialist care is available. This protocol is also communicated to pediatricians and emergency departments throughout the province, ensuring a coordinated and rapid response to any complications.

### 4.5. Limitations and Future Directions

This study has several limitations. As a retrospective analysis, it is subject to potential biases related to patient selection, incomplete documentation, and loss to follow-up. Moreover, the primary focus was on perioperative complications—especially hemorrhage—rather than on long-term functional outcomes.

The use of the Colorado microdissection needle, standardized perioperative protocols, and structured psychological support for families may have contributed to the favorable outcomes observed in our high-volume network. However, the retrospective, non-comparative design precludes any causal inference. No internal comparator group using alternative techniques (such as cold steel tonsillectomy) was available, and important sources of confounding—including surgeon experience, patient selection, referral pathways, and unrecorded changes in practice over two decades—could have influenced the results. Therefore, the associations reported in this study should be interpreted as exploratory and hypothesis-generating.

Additionally, pooling data across two decades (2005–2024) into a single cohort analysis risks masking secular trends or changes in practice. Over this period, unmeasured changes likely occurred in anesthesia protocols, postoperative analgesia, surgical training, potential underreporting of complications (residual risk of underreporting for hemorrhages presenting to non-Modena hospitals, though minimized by regional referral patterns and cross-verification with emergency databases, estimated capture > 95%) and the lack of internal control group. These temporal variations represent a limitation of our analysis, as we did not perform temporal stratification of outcomes.

Postoperative antibiotic use followed local practice patterns over the 20-year period and was not standardized or prospectively evaluated, which limits any interpretation of its potential impact on infectious or hemorrhagic outcomes.

Future prospective, multicenter studies with standardized follow-up protocols are needed to provide more robust evidence on the long-term effectiveness and safety of pediatric adenotonsillectomy. Further research should also address patient-reported outcomes, including sleep quality, daytime functioning, hearing improvement, and reduction in infection frequency, as well as quality-of-life measures for both patients and caregivers. Finally, comparative studies evaluating different surgical techniques, including the Colorado microdissection needle and other energy-based devices, would help clarify their relative impact on postoperative pain, bleeding, and regrowth rates.

## 5. Conclusions

Our multicentric 20-year experience within the hub-and-spoke structure of the Modena province, with Sassuolo Hospital as a central hub for medium- and low-complexity pediatric otolaryngology, while the University Hospital of Modena provides essential 24/7 tertiary-level support for complex and emergency cases, demonstrates favorable outcomes with complication rates at the lower end of published benchmarks. This territorial model appears effective for delivering safe pediatric adenotonsillectomy care, supported by high volume, innovative techniques, and integrated emergency pathways. Future prospective studies are warranted to validate these observations.

## Figures and Tables

**Table 1:** Postoperative hemorrhage requiring revision: stratification by anatomical origin (adenoidectomy vs. tonsillectomy-related; OR vs. tonsillectomy) and tonsillar technique (intracapsular vs. extracapsular; OR vs. extracapsular). ORs with 95% CI; χ^2^ test (*p* < 0.05 significant).

Characteristic	Revision (*n* = 75)	OR (95% CI) vs. Reference	*p*-Value
Anatomical origin			
Adenoidectomy (reference)	12	1	-
Tonsillectomy-related	63	8.25 (2.95–23)	<0.001
Tonsillar technique (*n* = 63)			
Extracapsular (reference)	52	1	-
Intracapsular	11	0.78 (0.37–1.65)	0.41

**Table 2:** Postoperative hemorrhage stratification by surgical indication (recurrent tonsillitis vs. OSA; OR vs. OSA reference; otitis media vs. OSA; OR vs. OSA reference) and age group for surgical revisions (3–5 years vs. >10 years; OR vs. 10 years reference). ORs with 95% CI; χ^2^ test (*p* < 0.05 significant).

Risk Factor	Hemorrhage Rate (%)	OR (95% CI) vs. Reference	*p*-Value
Indication			
OSA (reference)	1.63 (85/5215)	1	-
Recurrent tonsillitis	2.28 (95/4158)	1.41 (1.05–1.89)	0.021
Otitis media	1.59 (22/1380)	0.98 (0.62–1.54)	0.78
Age group (revisions)			
>10 years (reference)	20% (15/75)	1	
6–10 years	29.3% (22/75)	1.6 (0.8–3.2)	0.18
3–5 years	50.7% (38/75)	2.1 (1.1–4)	0.02

**Table 3:** Comparative data between the Colorado microdissection and conventional electrocautery. Data compiled from the published literature and institutional series.

Parameter	Colorado Microdissection Needle	Conventional Electrocautery
Intraoperative blood loss	5–20 mL	30–70 mL
Average operating time	22–30 min	25–35 min
Postoperative pain (day 1–5)	20–30% lower VAS score	Higher VAS score
Thermal damage	<0.7 mm spread	Up to 2 mm lateral spread
Postoperative hemorrhage rate	0.8–1.5%	2–4%

## Data Availability

The original contributions presented in this study are included in the article. Further inquiries can be directed to the corresponding author.

## Abbreviations

Obstructive sleep apnea (OSA), post-anesthesia care unit (PACU), otitis media with effusion (OME), standard deviation (SD), odds ratio (OR), confidence interval (CI), Visual Analog Scale (VAS).
